# Supplementary material for: Human-derived microRNA 21 regulates indole and L-tryptophan biosynthesis transcripts in the gut commensal Bacteroides thetaiotaomicron
Source: mBio. 2025 Jan 29;16(3):e03928-24. doi: 10.1128/mbio.03928-24 (PMC11898669; doi:10.1128/mbio.03928-24)
Supplement: Supplemental Figures — Figures S1 to S4. [file mbio.03928-24-s0001.pdf]

Supplementary Information

Human-derived microRNA 21 regulates indole and L-tryptophan biosynthesis transcripts in the gut commensal *Bacteroides thetaiotaomicron*

Kayla Flanagan[1], Kirsten Gassner[1], Michaela Lang[1], Jurgita Ozelyte[1], Bela Hausmann[2,3], Daniel Crepaz[1], Petra Pjevac[1,2], Christoph Gasche[4], David Berry[1,2], Cornelia Vesely[5#] and Fatima C. Pereira[1,6#]

This file contains: Supplementary Figures 1 to 4.

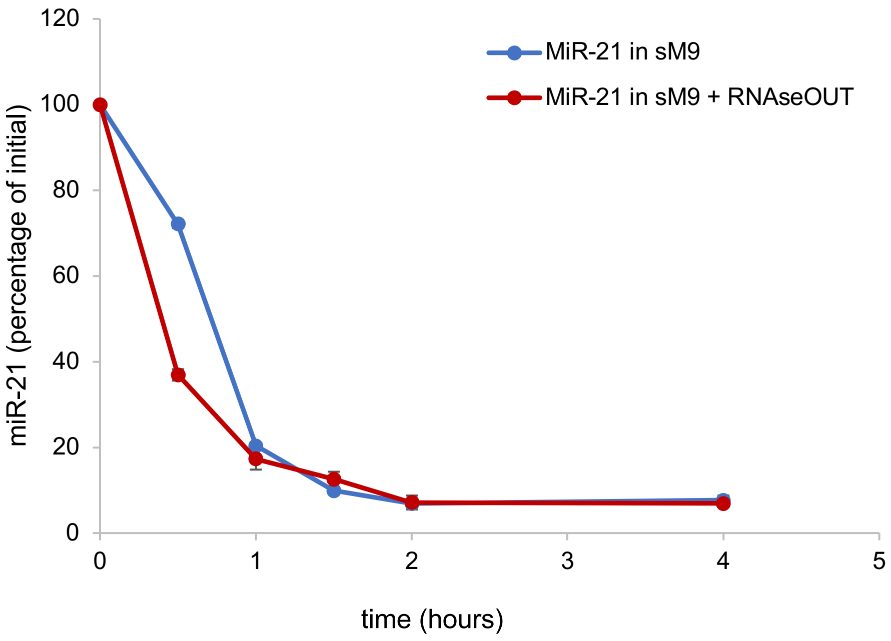

Figure S1. Impact of RNAse treatments on miRNA association dynamics. Percentage of the initial miRNA measured concentration remaining over time in live cell incubations with miR-21 alone versus miR-21 in combination with an RNase inhibitor (RNAseOUT). Data points represent the mean ± standard deviation of two replicates.

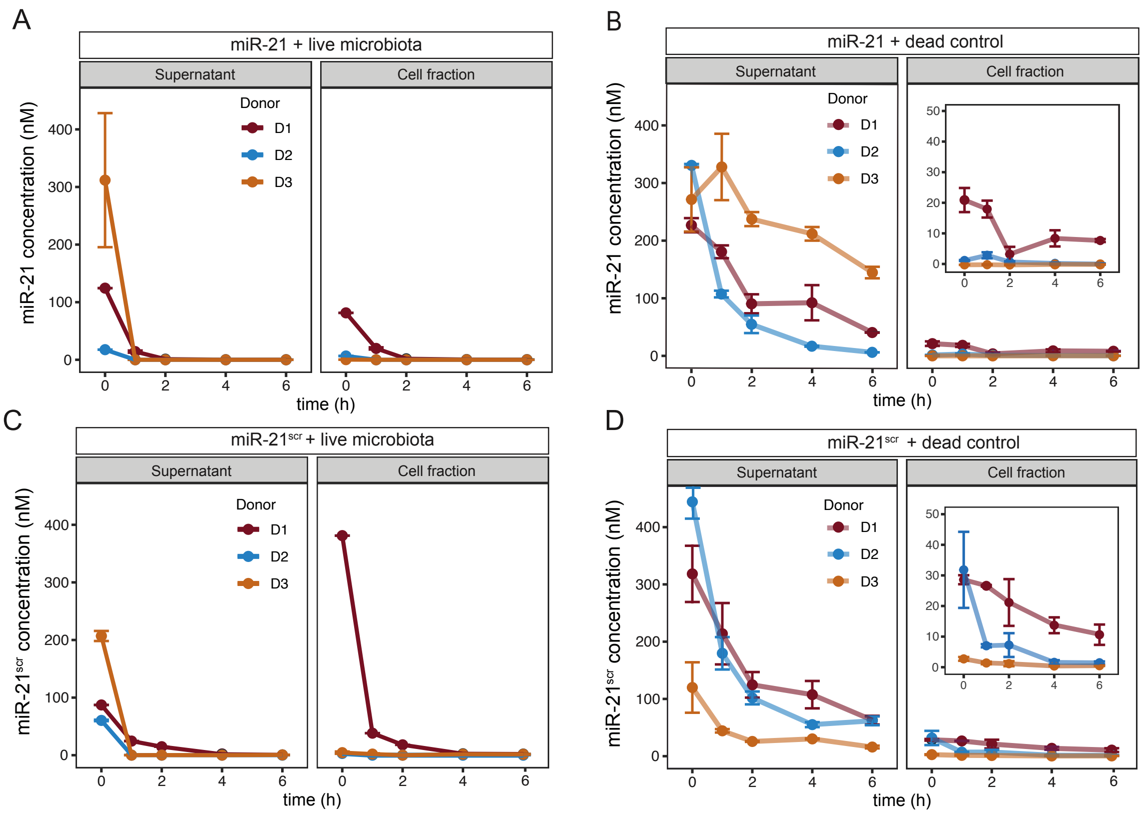

Figure S2. Quantification of miRNAs in faecal samples and dynamics of association of miRNAs with faecal microbiota. A, B. Concentrations of miR-21 over time in live (A) or dead control (B) microbiota incubations amended with miR-21, determined by qPCR. Three independent sets of incubations were established using faecal samples from three healthy donors (D1, D2, D3). **C, D.** Concentrations of miR-21^{scr} over time in live (C) or dead control (D) incubations amended with miR-21^{scr}, for the same three donors. Data points represent the mean of two replicates per condition and bars represent the standard deviation.

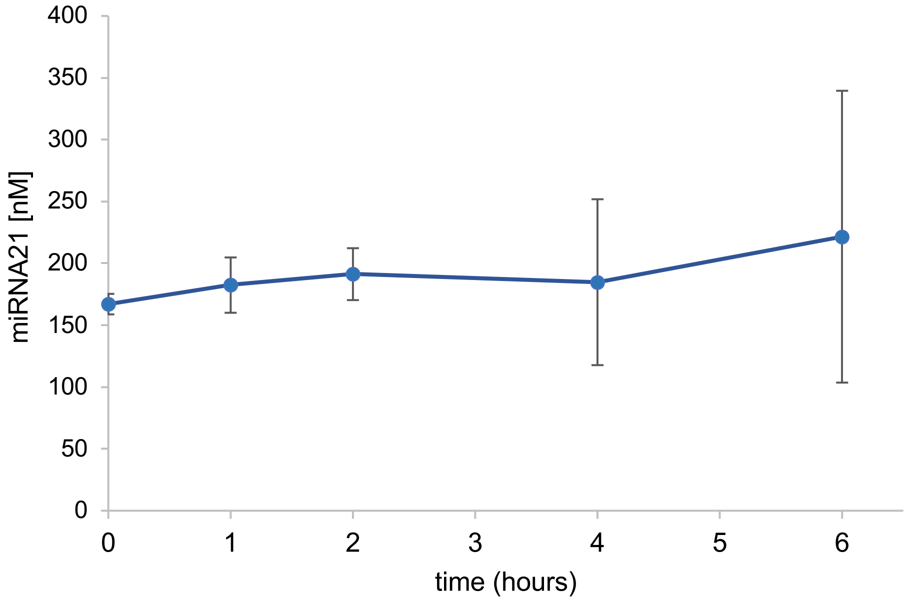

Figure S3. Stability of miR-21 mimics in incubation vials. Change in miR-21 concentration over time in incubation medium alone (sM9), as determined by qPCR. Data points represent the mean ± standard deviation of two replicates.

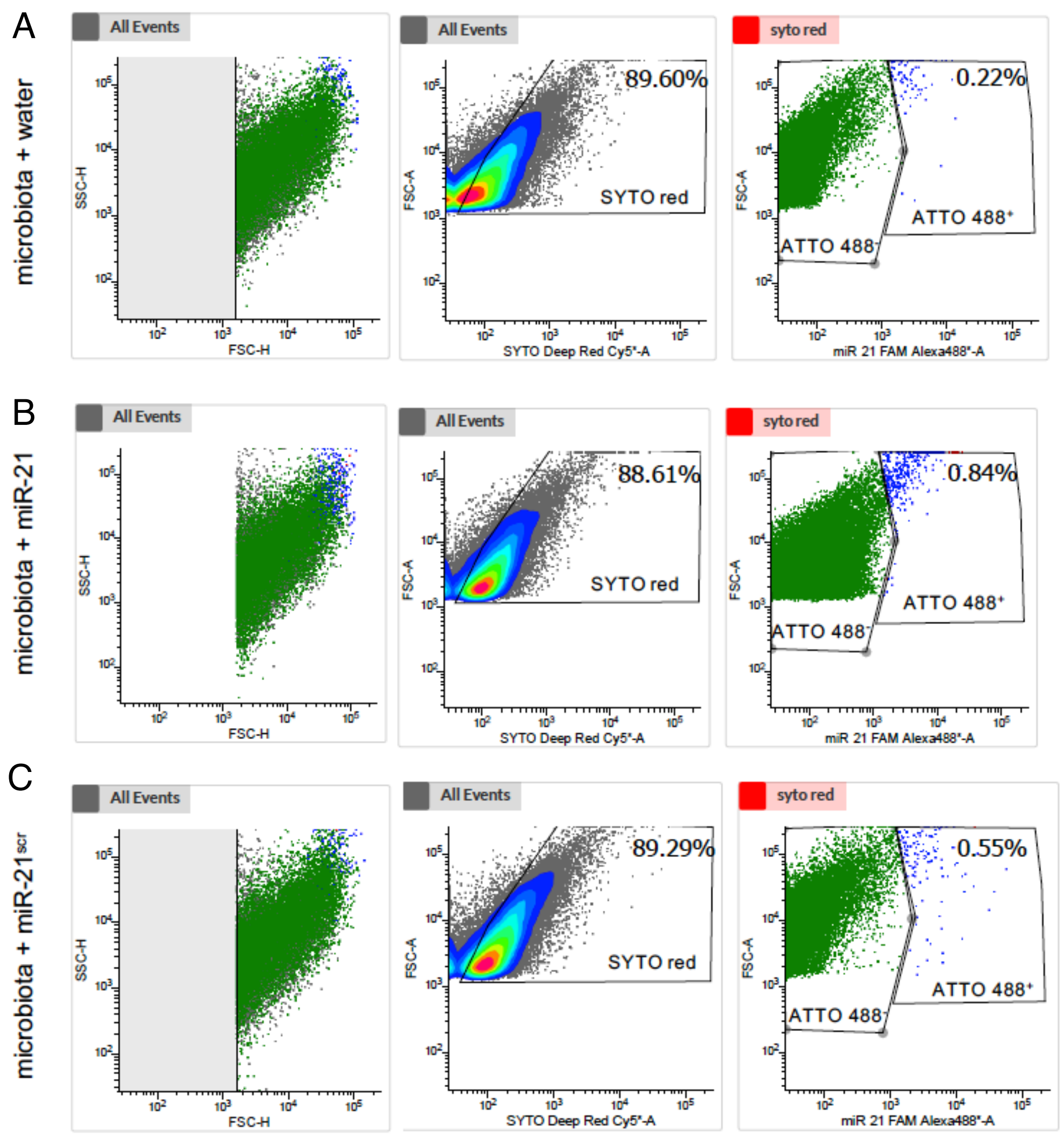

Figure S4. Flow cytometry gating strategy to sort cell events interacting and not interacting with fluorescently labelled miRNAs. A fixed staining-gating strategy to sort faecal sample cell events supplemented with water (**A**), ATTO488-labelled miR-21 (**B**), or ATTO488-labelled miR-21^{scr} (**C**) is shown. Left panels show forward and side scatter plots with all events. Middle panels show a density plot with gating of SYTO red^{+} cells (SYTO 62 red staining), to distinguish cells from debris and background noise. Percentages of SYTO red^{+} events are indicated. Left panels show gating of ATTO 488^{+} and ATTO 488^{-} signal, which was performed using Alexa488 channel to discriminate cell events (SYTO red+) displaying or not ATTO 488^{+} signal. The panels on the right also indicate the percentage of cells falling into the ATTO 488^{+} and ATTO 488^{-}. The acquisition rate did not exceed 2,000 events per second. Each panel reflects over 100,000 registered events.
